# Widespread adoption of environmental DNA (eDNA)-based biodiversity monitoring in Canada will depend on trust more than on education: insights from a SWOT analysis

**DOI:** 10.1007/s10661-026-15262-9

**Published:** 2026-03-28

**Authors:** Caroline Thivierge, Limoilou-Amélie Renaud, Jérôme Dupras, Caren C. Helbing, Valerie S. Langlois, Hugo Asselin

**Affiliations:** 1 Department of Natural Sciences, Université du Québec en Outaouais, Gatineau, Canada; 2 Canada Research Chair in Ecological Economics, Montréal, Canada; 3 School of Indigenous Studies, Université du Québec en Abitibi-Témiscamingue, Rouyn-Noranda, Québec Canada; 4 Chaire Desjardins en Développement Des Petites Collectivités, Université du Québec en Abitibi-Témiscamingue, Rouyn-Noranda, Canada; 5 Department of Biochemistry and Microbiology, University of Victoria, Victoria, BC Canada; 6 Centre Eau Terre Environnement, Institut National de La Recherche Scientifique (INRS), Quebec City, QC Canada

**Keywords:** Environmental DNA, SWOT analysis, Innovation adoption, Mixed methods, Social acceptability, Power relations, Trust

## Abstract

The rapid decline in biodiversity underscores the urgent need for robust monitoring systems to assess ecological responses to environmental change and to inform effective conservation and sustainable management strategies. Owing to its non-invasive nature, high sensitivity, rapid results, and cost-effectiveness, environmental DNA (eDNA) represents a promising approach to address this challenge. Nevertheless, the limited uptake of eDNA beyond scientific contexts suggests that the characteristics of the innovation alone are insufficient to ensure its adoption. With this study, we aimed to (i) compare the perceptions of eDNA experts and end-users about the capabilities of eDNA-based monitoring methods, (ii) identify the strengths, weaknesses, opportunities, and threats (SWOT) associated with the use of eDNA for biodiversity monitoring in Canada, and (iii) examine whether differences in perceptions stem from socio-demographic factors. We used SWOT analysis to identify key internal and external factors influencing eDNA uptake across stakeholder groups. Our findings reveal broad consensus regarding the strengths and opportunities of eDNA for biodiversity monitoring, alongside more heterogeneous views on its limitations and implementation challenges, while socio-demographic variables showed limited influence on perception patterns. Trust emerged as a pivotal factor influencing the adoption of eDNA-based monitoring, encompassing multiple dimensions: confidence in protocols, skepticism toward laboratories and regulatory bodies, concerns over user capacity, and apprehensions regarding the interpretation and potential misapplication of results. To broaden eDNA uptake in Canada, we recommend emphasizing not only education, but also alignment with end-user expectations to increase confidence in the methods, and trust among organizations.

## Introduction

The accelerated decline of biodiversity worldwide represents an increasing threat to ecosystem stability and human well-being (Bennett et al. 2015; Cardinale et al. 2012). Effective biodiversity monitoring is essential to understand the consequences of environmental changes and guide conservation and sustainable management actions (Noss, 1990; Stephenson et al., 2022; Urbano et al., 2024). To that end, it has been suggested that environmental DNA (eDNA), which refers to genetic material shed by organisms into the environment (i.e., water, soil, air), is a promising tool to facilitate biodiversity monitoring (Ficetola et al., 2008; Taberlet et al., 2012). eDNA analysis encompasses a suite of laboratory and analytical methods, including targeted (e.g., qPCR) and untargeted (e.g., metabarcoding) techniques. Monitoring methods based on eDNA can save time and costs compared to conventional approaches (Ruppert et al., 2019; Van Klink et al., 2022). Indeed, eDNA sampling is rapid and easy (Doi et al., 2017) and the costs of eDNA analyses are constantly decreasing as technology improves (Harper et al., 2018). Contrary to conventional monitoring methods (e.g., seining, trapping, bottle traps, acoustic detection) which often focus on a single or a small group of species (Swan et al., 2014) or can miss inconspicuous species (Deacon et al., 2017), eDNA can detect more species (Fediajevaite et al., 2021) and it can provide a full biodiversity scan if metabarcoding methods are used (Afzali et al., 2021; Ruppert et al., 2019). Metabarcoding is also useful for environmental impact assessments (e.g. Allan et al., 2023; Everts et al., 2024; Xu et al., 2023).

The benefits of eDNA over conventional biodiversity monitoring methods and the challenges associated with eDNA use have been covered elsewhere (Allen et al., 2021; Beng & Corlett, 2020; Blanchet, 2012; Everts et al., 2024; Rees et al., 2014; Ruppert et al., 2019; Xu et al., 2023). Briefly, eDNA allows species detection at low abundance levels (Allen et al., 2021), is non-invasive (Kelly et al., 2014), yields rapid results to facilitate timely decision-making (Darling & Mahon, 2011), can be cost-effective (Evans et al., 2017; Ruppert et al., 2019; Veldhoen et al., 2016) and is especially useful in data-sparse settings (McClenaghan et al., 2020).

Despite its advantages, eDNA faces limitations and ongoing challenges that hamper its broad application across ecological contexts. One major constraint has been the lack of reliable models for estimating relative abundance or biomass from eDNA data, which limits its utility for population assessments (Biggs et al., 2015; Bohmann et al., 2014). Although recent developments have enabled quantitative estimates of fish abundance in certain contexts (Shelton et al., 2022; Su et al., 2024), these approaches remain challenging and are far from universally applicable. Additionally, while eDNA has shown promise for broadening participation in biodiversity monitoring through citizen science initiatives (Biggs et al., 2015; Larson et al., 2020), ensuring data quality and standardization remains a challenge in such approaches (Bohmann et al., 2014). Moreover, eDNA often fails to provide information on life-history stages, although new eRNA-based methods show some promise (Parsley & Goldberg, 2024), and interpreting presence data without insight into organism abundance, seasonality, or activity can lead to misinformed conclusions (De Souza et al., 2016; Goldberg et al., 2016). For metabarcoding approaches specifically, detection probability remains poorly understood and difficult to quantify (Wilcox et al., 2016), and taxonomic detection biases—most notably favoring vertebrates over other taxa (Tréguier et al., 2014)—may distort biodiversity assessments. These challenges are compounded by persistent limitations in reference databases, including incomplete taxonomic coverage and uneven resolution, as well as the absence of standardized protocols for field sampling and laboratory analyses (Keck et al., 2022). Recent initiatives, such as those led by the Canadian Standards Association (CSA), are beginning to address these gaps (CSA Group, 2021, 2023).

While eDNA is a powerful tool, its performance remains highly context-dependent, showing greatest success in aquatic environments, including sediments (Huston et al., 2023), but limited application in terrestrial (Allen et al., 2021; Matthias et al., 2021) and aerial settings (e.g., Clare et al., 2021). Beyond practical and technological limitations, the adoption of eDNA faces notable social and institutional barriers (Doi & Nakamura, 2023; Hansen et al., 2018). There are ongoing debates about the strengths and limitations of eDNA. Moreover, eDNA methods require specialized facilities, substantial computational resources, and trained personnel (Yoccoz, 2012), hindering broad adoption. Paradoxically, although eDNA is often considered more sensitive than conventional monitoring methods, results are frequently validated against these traditional approaches (Darling, 2015), which reflects a lack of trust in eDNA methods, particularly among non-academic stakeholders (Darling, 2019; Lee et al., 2023).

Currently, the use of eDNA methods is largely concentrated within academic institutions (Petruniak et al., 2021). Its dissemination to other stakeholder groups – including regulatory bodies, consulting firms, local communities, and non-governmental organizations – is taking comparatively more time (Kelly et al., 2023). Limited uptake may reflect both organizational constraints (e.g., lack of technical capacity, time, or funding) and epistemic concerns, including uncertainty about protocols and lack of trust in the results (Doi & Nakamura, 2023; Lee et al., 2023). Moreover, the growing number of private companies in this field may amplify trust issues as they often have limited experience and may oversell their capabilities (Shen et al., 2024).

Not all potential end-users are equally comfortable adopting or interpreting eDNA data (Burian et al., 2021; Hansen et al., 2018). In many cases, the limitations and uncertainties of eDNA are not well understood outside academic circles, making it difficult for non-specialists to critically evaluate the results or account for potential biases (Burian et al., 2021; Lortie & Owen, 2020). This lack of understanding may lead to overconfidence in eDNA outputs or, conversely, skepticism depending on how the results are communicated. Various initiatives have promoted eDNA through blanket communication strategies, without adequately consulting stakeholders about their needs, expectations, or reservations (Petruniak et al., 2021). Addressing these social dimensions – by fostering transparent communication, offering targeted training, and developing institutional structures – is essential for building the legitimacy and usability of eDNA as a decision-support tool.

The purported benefits of an innovation do not guarantee its adoption by new end-users. The adoption – or not – of an innovation depends on perceptions of usefulness, ease of use (Davis, 1989; Hess et al., 2014; Yousafzai et al., 2007), perceived risk and trustfulness (Pavlou, 2003), which can be influenced by a multitude of factors such as individual beliefs (Venkatesh, 2000), social norms (Irimia-Diéguez et al., 2023; Schultz et al., 2007), expertise (Taylor & Todd, 1995), and experience (Bandura, 1986), as well as the social environment (Talukder & Quazi, 2011). How a message is communicated, along with the perceived credibility of the diffusion agent, significantly influences the adoption decision (Rogers, 2003). This underscores the pivotal role of trust, both in expected outcomes and in the relational dynamics between individuals and institutions. Limited understanding of the methodology, its constraints and potential applications, may negatively affect perceived usefulness and reduce trust in eDNA methods (Thivierge et al., 2025). While knowledge gaps can be addressed through training initiatives, it is also essential to understand the concerns of various stakeholder groups with eDNA methods, some due to power imbalances, which might hinder broader adoption. Mechanisms can then be implemented (regulations, norms) to allay suspicion. Comparing the perceptions of end-users with the views of eDNA experts can help differentiate between misconceptions and evidence-based assessments. It can also reveal divergences among expert opinions that may lead end-users to perceive the approach as immature or misaligned with their needs.

The objectives of this study were threefold: (i) to compare the perceptions of eDNA experts and end-users about the capability of eDNA-based monitoring methods; (ii) to identify the strengths, weaknesses, opportunities, and threats (SWOT) associated with the use of eDNA for biodiversity monitoring in Canada; and (iii) to examine whether differences in perceptions stem from socio-demographic factors. The SWOT framework allowed us to separate intrinsic properties of eDNA methods (strengths and weaknesses) from external conditions shaping their uptake (opportunities and threats), offering a strategic overview of adoption barriers and leverage points complementary to innovation-diffusion approaches. To our knowledge, this is the first in-depth SWOT analysis of eDNA adoption across user groups in Canada to integrate end-users’ perceptions and experts’ insights.

## Material and methods

### Elaboration of the SWOT questionnaire

**Fig. 1:**
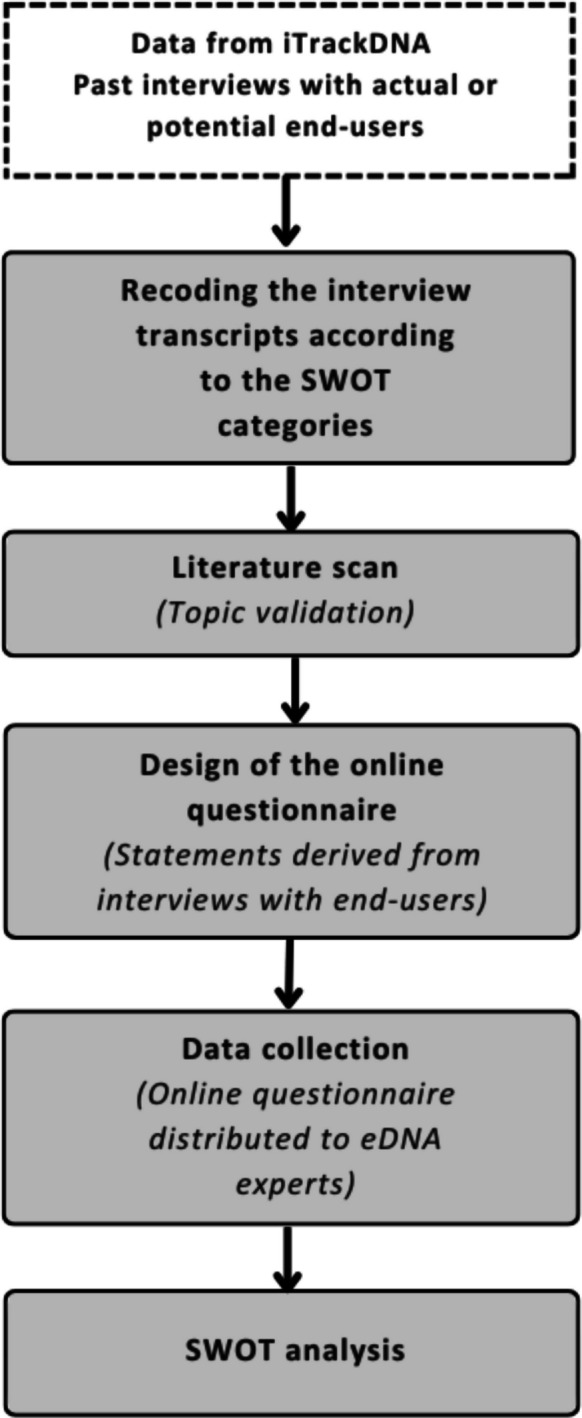
SWOT analysis development process

Figure 1 illustrates the process used to develop the SWOT analysis. For this study, we relied on secondary data from two series of interviews previously conducted with potential and current eDNA end-users (n = 61; 49% women, 51% men) as part of other projects within the iTrackDNA initiative (https://itrackdna.ca/). The interviewed end-users included environmental non-governmental organization (ENGOs) (n = 16), regional administrators (n = 3), private sector (n = 9), academics (n = 7), and government affiliated scientists (n = 10) or managers (n = 8), as well as individuals (n = 3) and groups who engage with the land for cultural or subsistence reasons, such as members of Indigenous communities (n = 5). The original purpose of these interviews was to explore end-users’ perceptions of eDNA, specifically its perceived usefulness, ease of use, and compatibility with their activities, in order to identify potential drivers and barriers to its adoption. For the present study, the interview transcripts were recoded and reanalyzed using the MaxQDA software (version 24.8.0; VERBI Software GmbH, Berlin, Germany) to generate statements related to strengths, weaknesses, opportunities, and threats associated with the use of eDNA for biodiversity monitoring. We then scanned the literature to identify additional topics that might not have been mentioned in the interviews. The literature scan confirmed that most relevant topics had been addressed in the interviews. The literature scan also helped us synthesize interview contents into coherent statements. To ensure representativeness, only perspectives shared by more than one end-user were included and we made sure that no rogue or outlier statement was included. Certain topics appear under both strengths and weaknesses (or opportunities and threats), such as cost and cost-effectiveness, to reflect divergent viewpoints among end-users. The statements were then arranged into an online questionnaire which we distributed to a panel of eDNA experts from the iTrackDNA network (members and collaborators) to assess their alignment with end-users' perceptions. We generated 82 statements organized into the four SWOT categories, and six additional statements related to trust toward eDNA-based monitoring methods (as trust was a major issue revealed by our literature scan). The statements were used to construct a questionnaire with 19 questions: 6 socio-demographic, 5 multiple-choice (including the statements), and 8 open-ended. The multiple-choice questions followed a Likert scale with four options (strongly disagree, disagree, agree, and strongly agree), with no neutral option to compel respondents to take a position. The open-ended questions allowed respondents to provide additional insights or raise issues not explicitly addressed in the questionnaire’s statements.

### Data collection

A targeted sample of 60 eDNA experts affiliated with the iTrackDNA initiative was initially contacted to complete the questionnaire. This initial sampling was further complemented by a snowball sampling approach, whereby eDNA experts were encouraged to disseminate the study among their professional networks. A total of 38 eDNA experts responded to the questionnaire. We used Microsoft Forms (Microsoft Corporation, Redmond, WA, United States) to distribute the questionnaire to the respondents, who had four weeks to reply, from March 6 to April 4, 2025. The average completion time was 43 min.

### Analytical process

We used the R software (version 4.4, R Core Team, 2025) to analyze survey responses. We calculated the percentage of respondents who expressed agreement with each statement. We then ranked statements from the most to the least supported within each SWOT category. Responses 'agree' and 'strongly agree' were grouped and classified under 'agreement,' while 'disagree' and 'strongly disagree' were grouped and classified under 'disagreement.' This categorization was employed to capture the overall direction of eDNA experts' attitudes while preserving the distinction between general agreement and stronger levels of endorsement or opposition. The percentage of agreement was calculated by dividing the number of respondents who agreed with a given statement by the total number of respondents who answered that statement. The open-ended responses were analyzed using the MaxQDA software. Codes were grouped by thematic similarity, and the number of respondents who reported each theme was noted.

For statements with over 60% agreement, we interpreted experts’ opinions as being aligned with end-users’ perceptions of eDNA. For statements with less than 40% agreement, we considered that experts’ and end-users’ opinions differed. Statements with intermediate agreement (40–60%) were interpreted as reflecting mixed opinions among experts, some agreeing and some disagreeing with end-users. These thresholds were chosen to distinguish broad consensus from heterogeneous responses. We assessed the robustness of this analysis by applying a more liberal disagreement range (30–70%), but it did not alter the overall patterns and conclusion.

## Results

The results are presented in the following order: the socio-demographic characteristics of the respondents, followed by findings organized according to the SWOT categories. Each subsection begins with the quantitative results, followed by the findings from the open-ended questions and by the exploration of patterns of variation among respondents for statements that received mixed agreement.

### Respondent socio-demographics

**Fig. 2:**
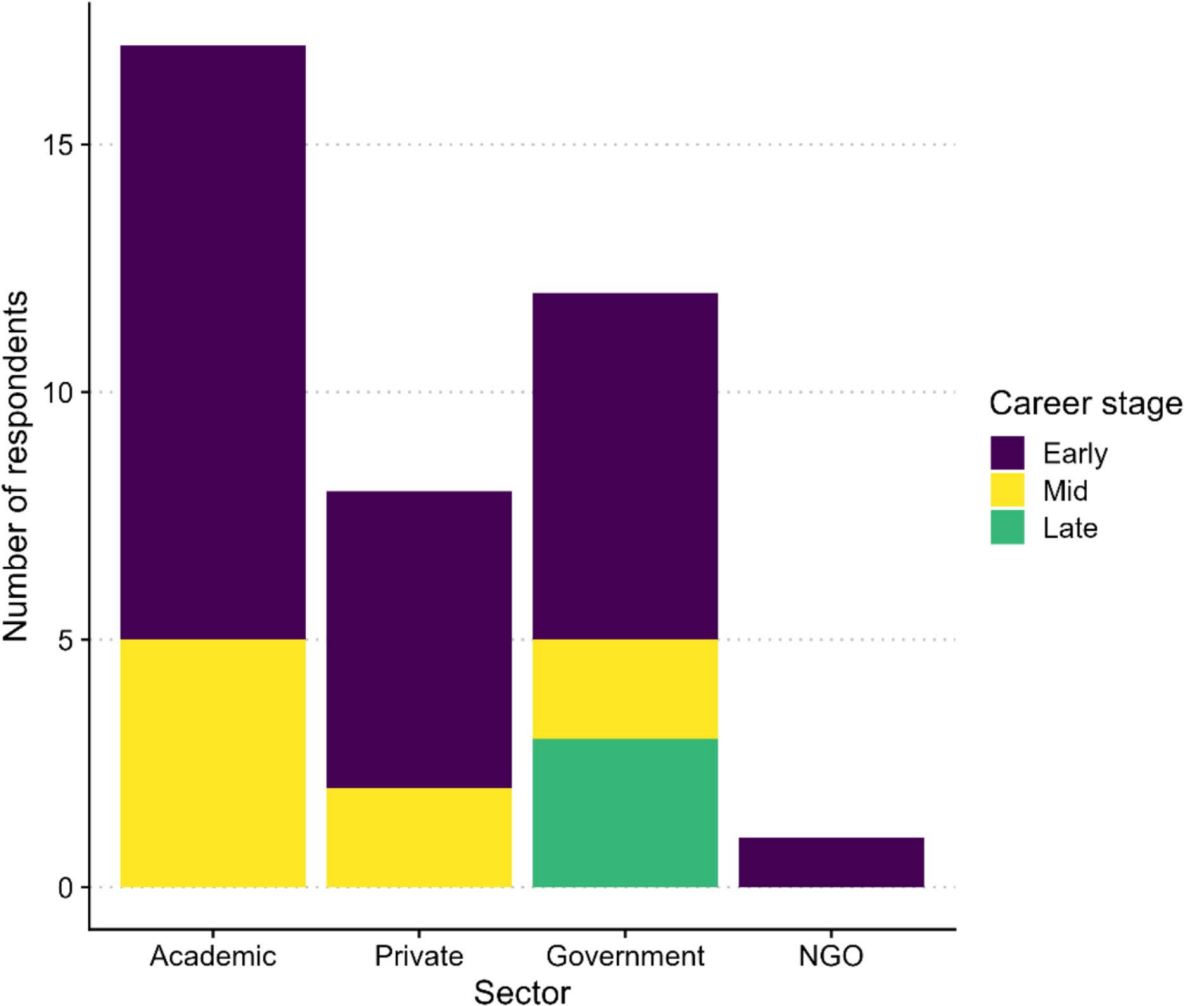
Number of respondents and their career stage. Early career was defined as less than 10 years of experience; mid-career as 10 to 20 years of experience and late career as more than 20 years of experience

A total of 38 individuals responded to the questionnaire, 21 (55%) were women and 17 (45%) were men, 22 were from four provinces and one territory in Canada and the others (16) were from the United States of America and Europe (Fig. 2). It was not possible to accurately identify the country of origin of European respondents, as the questionnaire was not initially designed for international use. The 16 respondents from outside Canada were invited to participate because they collaborate with scientists in Canada and are therefore familiar with the Canadian context. Respondents included 23 researchers, 6 research professionals and technicians, 6 graduate students and postdocs, and 3 managers. They were employed across 17 positions in academia, 12 in the public sector, 8 in the private sector, and 1 in a non-governmental organization (NGO).

### Strengths

**Fig. 3:**
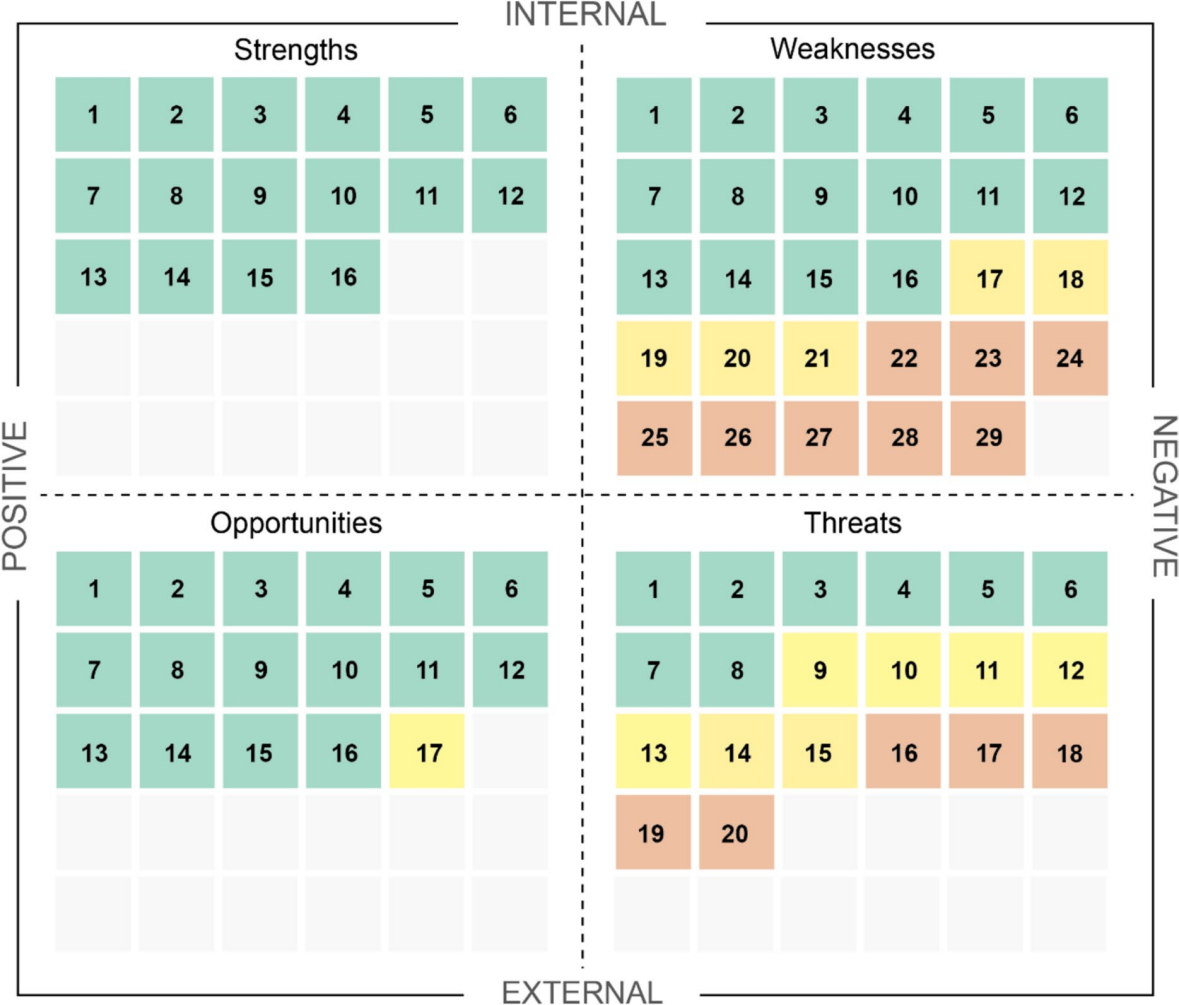
Summary results of the SWOT analysis of perceived benefits and limitations of environmental DNA (eDNA) adoption. Each quadrant represents one SWOT dimension—Strengths and Weaknesses (internal factors), Opportunities and Threats (external factors). Numbered squares refer to specific survey statements (Tables 1, 2, 3 and 4). Colors indicate the levels of agreement with the statements: green (> 60% of agreement), yellow (40–60%), and orange (< 40%)

Respondents showed near-unanimous agreement on the core strengths of eDNA for biodiversity monitoring, particularly its ability to detect a wide range of taxa, including rare, elusive, and invasive species, while supporting decision-making and minimizing environmental disturbance. Agreement was slightly more moderate regarding economic benefits, the role of non-scientists in sampling, and the perceived necessity to standardize procedures (Table 1). Figure 3 synthesizes the results of the SWOT analysis of perceived benefits and limitations of environmental DNA (eDNA) adoption, mapping survey statements onto the four SWOT categories and displaying levels of agreement among respondents.

**Table 1:** eDNA expert agreement rates with statements related to eDNA strengths generated from interviews with end-users

Statement ID	Survey statement	Agreement with statement (% of total respondents)
S1	eDNA can provide the basis for a decision to deploy a field team for more in-depth analysis	100
S2	eDNA can detect a wide variety of species, even those difficult to observe directly	100
S3	eDNA is effective for detecting endangered species	94.7
S4	eDNA is effective for detecting species at low abundance	94.7
S5	eDNA makes it possible to monitor the appearance and spread of invasive exotic species effectively and at an early stage	94.7
S6	eDNA is particularly useful for measuring biodiversity in difficult-to-access areas	94.7
S7	eDNA provides information on flora and fauna without disturbing the natural environment, whether in accessible or inaccessible sites	94.7
S8	eDNA can support decision-making processes relating to rare, threatened, and invasive species	94.7
S9	Approaches based on eDNA can support environmental impact assessments	94.7
S10	The use of eDNA makes it easier to conduct population inventories frequently and at short time intervals (e.g. annual sampling rather than every five or ten years)	92.1
S11	eDNA is relevant for habitat monitoring (e.g., after logging, at a restored mine site, within a permanent inventory or research plot, etc.)	86.8
S12	eDNA is a time-saving method compared to conventional inventory methods	84.2
S13	eDNA sampling can be carried out by non-scientists	81.6
S14	The requirement to follow the CSA standard is essential to ensure comparability of data between different laboratories	76.3
S15	The use of eDNA leads to cost savings	71.1
S16	The CSA standard guarantees the reliability and quality of eDNA results	68.4

Two open-ended questions allowed respondents to identify what they believe is the main strength of eDNA compared to conventional approaches and whether they attribute other strengths to eDNA-based methods that were not addressed in the questionnaire. Among the most frequently mentioned advantages (n = 2 or more occurrences) were: less invasive approach (n = 8), ease of use (n = 8), sensitivity (n = 7), especially for species that are rare, at low abundance or at early stages of development, precision (n = 2), and the fact that it reduces observer bias (n = 2). With regard to other strengths not addressed in the questionnaire, respondents mentioned that eDNA is not a time-constrained method (i.e., sampling is possible outside the usual monitoring periods), and the possibility of retroactive testing or re-using a sample at any time, even years later, to test for the presence of other species. The latter strength was said to be a major advantage compared with conventional approaches.

### Weaknesses

Agreement on the weaknesses of eDNA-based monitoring methods was less pronounced than for strengths, although respondents did converge on many key limitations (Table 2). The highest agreement concerned inherent methodological constraints and expertise requirements, whereas more mixed views emerged for issues related to training, cost, and technical development.

**Table 2:** eDNA expert agreement rates with statements related to eDNA weaknesses generated from interviews with end-users

Statement ID	Survey statement	Agreement with statement (% of total respondents)
W1	Equipment (sequencers) must be maintained by professionals	100
W2	The absence of DNA detection in a sample does not necessarily mean the absence of the species in the environment	97.4
W3	The information obtained by eDNA does not allow us to know the health status of the targeted species	92.1
W4	Only qualified personnel can perform eDNA analyses in the laboratory	89.5
W5	Conception of experimental designs requires the support of an environmental professional	89.5
W6	eDNA does not accurately estimate the biomass of the targeted species at the sampled site	84.2
W7	eDNA does not accurately estimate the abundance of the targeted species at the sampled site	81.1
W8	Potential end-users are not sufficiently equipped to interpret eDNA sampling results	78.9
W9	Many samples are needed to ensure environmental representativeness	76.3
W10	eDNA cannot replace conventional methods of inventorying wildlife populations	71.1
W11	The total cost of an eDNA study is high (primer design, bioinformatics analyses, etc.)	71.1
W12	Potential end-users of eDNA are not sufficiently informed about the technique to use it properly	68.4
W13	False negatives are common in eDNA analysis	63.2
W14	Reference databases are insufficient to meet current needs	63.2
W15	The cost of acquiring eDNA analysis equipment hinders the spread of the approach to other end-users	63.2
W16	Analysis results are not disseminated by laboratories before being sent to customers	60.5
W17	There are not any training courses in the use of eDNA available to everyone	59.5
W18	Many samples are needed to ensure valid results	52.6
W19	The detection of DNA does not confirm the presence of the target species in the sampled area	50
W20	The development of specific primers and tests is an obstacle to the use of eDNA	47.4
W21	Access to specialized equipment is a major obstacle to the use of eDNA	42.1
W22	The application of experimental design is too complex for non-scientists	39.5
W23	My organization does not carry out any knowledge transfer activities on eDNA accessible to all	38.9
W24	There is an insufficient number of primers and specific tests for the species I want to study or monitor	36.8
W25	False positives are common in eDNA analysis	28.9
W26	eDNA sampling carries more risk of error than conventional data collection (e.g. bird counts by observation or listening)	26.3
W27	Sample handling, transport and storage are too complex for non-scientists	21.6
W28	eDNA is too expensive to be used at a large scale	18.4
W29	eDNA sampling is not relevant for temporal monitoring of wildlife species or habitats	13.2

Three open-ended questions were asked: (1) to mention other weaknesses of eDNA-based methods that were not addressed in the questionnaire, (2) to identify the main limitations of using eDNA, and (3) to estimate the frequency of errors across different sampling environments, namely terrestrial, aquatic, and aerial.

According to the respondents, at least seven additional weaknesses of eDNA were identified that were not addressed in the questionnaire. Among the most frequently mentioned were the effect of eDNA transport and degradation on results (n = 6), the lack of clear criteria for interpreting positive and negative results (n = 4), the multiplicity and complexity of protocols (n = 4), the lack of standardization (n = 2), inaccurate or incomplete reference databases (n = 2), the context-dependence of eDNA-based estimates of abundance and biomass (n = 2), and the environmental consequences of single-use consumables (n = 2). Ten respondents indicated that they had no additional weakness to report beyond those covered in the questionnaire.

The respondents identified several limitations to the use of eDNA. Among the most frequently mentioned (reported by two or more respondents) were: the need for protocol standardization (n = 10), the inability to fully answer all research questions (n = 7), incomplete databases (n = 5), limited knowledge among end-users (n = 3), lack of proper assays (n = 3), the cost of eDNA-based methods (n = 3), inconsistent quality and reputation across laboratories (n = 2), limited availability or accessibility of laboratories (n = 2), and lack of training (n = 2).

Answers to the open-ended question regarding the frequency of detection of errors (false positives or false negatives) depending on the environment studied fell into three categories: a higher risk in terrestrial environments (n = 10), an equal risk regardless of the sampling environment (n = 9), and a higher risk in aerial environments (n = 5). Notably, eight respondents did not take a position, as they reported working exclusively in aquatic environments, which limited their ability to assess other environments. Four respondents emphasized that, in their view, the risk of error is not linked to the sampling environment, but rather to the initial experimental design, strict adherence to the sampling protocol, number of quality controls in the assays, and laboratory’s quality assurance system. Three respondents also noted that, in their opinion, false negatives are more frequent in terrestrial environments, whereas false positives are more common in aquatic environments.

### Opportunities

Overall, the respondents expressed strong consensus regarding the statements categorized as opportunities for the use of eDNA, notably its potential to enable monitoring across larger territories, the importance of knowledge transfer to foster wider adoption, and its role as a decision-support tool for land management (Table 3).

**Table 3:** eDNA expert agreement rates with statements related to eDNA opportunities generated from interviews with end-users

Statement ID	Survey statement	Agreement with statement (% of total respondents)
O1	Knowledge transfer activities about eDNA are essential to encourage its adoption by a wider audience	97.4
O2	Researchers have an important role to play in accompanying new end-users towards the interpretation of their results	97.4
O3	eDNA provides complementary information on biodiversity that cannot be obtained using other methods	94.7
O4	In the context of post-disturbance monitoring or restoration (e.g. a restored mining site), eDNA can guide decisions about land management	92.1
O5	Legal recognition of eDNA would encourage its use in conservation and environmental management projects	92.1
O6	eDNA allows sampling at a wider range of spatial scales than conventional inventory methods (e.g., larger territories, more isolated or less accessible sites)	89.5
O7	eDNA can guide choices of conservation actions	89.5
O8	Regulation of the use of eDNA would ensure more rigorous use of this technique	86.8
O9	Researchers specializing in eDNA collaborate with each other in the development of scientific knowledge	86.8
O10	I am confident in the results obtained by data collection carried out by citizens trained in eDNA use	81.6
O11	eDNA is suited to citizen participation in research or inventory projects	78.9
O12	The public funds and grants available encourage the transfer of knowledge to different audiences	78.9
O13	eDNA allows sampling at finer temporal scales and more frequently than conventional inventory methods	76.3
O14	The full potential of eDNA will be reached in a few years	71.1
O15	eDNA can be used as a unique approach to data collection	63.2
O16	eDNA can guide land management choices more accurately than conventional biodiversity sampling methods	60.5
O17	eDNA should be used exclusively as a complement to conventional inventory methods	55.3

The respondents were surveyed on what they consider to be emerging areas of eDNA application to enhance our ability to manage and conserve ecosystems. Their responses reflected a desire to address a broader range of research questions, including quantifying species abundance, sex, age structure, and both population-level and intra-specific genetic variation. New areas of application for eDNA were identified in the open-ended question. Among the most frequently mentioned (reported by two or more respondents) were: using eRNA to assess organism health and detect recent presence (n = 8), using eDNA to conduct environmental impact assessments (n = 6), performing population genetics across multiple species simultaneously (n = 4), estimating species abundance (n = 4), monitoring wildlife populations and supporting wildlife management (n = 3), paleoecology (n = 3), detecting sex and age structure (n = 2), and automated sampling and real-time results (n = 2).

### Threats

Responses regarding threats associated with eDNA-based approaches were more heterogeneous than those concerning opportunities, showing less overall convergence (Table 4). Respondents largely agreed with threats related to methodological and governance issues, including factors influencing detection probability, protocol standardization, data traceability, and access to publicly funded assays. Communication and knowledge gaps were also widely perceived as threats, while views were more mixed regarding laboratory capacity, commercialization, climate-change-related impacts, and public trust. Low agreement was expressed with issues such as environmental damage during sampling or climate-driven degradation of reference databases.

**Table 4:** eDNA expert agreement rates with statements related to eDNA threats generated from interviews with end-users

Statement ID	Survey statement	Agreement with statement (% of total respondents)
T1	The physical and chemical properties of sampled media (e.g., water, soil, air) affect the concentration of DNA and the probability of detection	97.4
T2	Publicly funded eDNA assays are not always freely available, and access may depend on the organization that created them	91.9
T3	Lack of sample traceability negatively affects the perceived reliability of results	89.5
T4	Disparities in the protocols used in different laboratories can call into question the comparability of results	89.5
T5	Information on the limits of eDNA is not sufficiently popularized to make it understandable to public decision-makers and end-users	76.3
T6	It is not always possible to determine the exact origin of an eDNA sample	70.3
T7	Current communication about eDNA is not enough to win public confidence	67.6
T8	An increase in demand for eDNA analysis could saturate laboratory capacity	64.9
T9	The limitations of eDNA are not sufficiently known to be considered in the interpretation of results by non-scientists	57.9
T10	Rapid improvement in eDNA analysis protocols compromises comparability of data over time	56.8
T11	Laboratory resources are insufficient to meet current demand	55.3
T12	Climate change could alter the quality of eDNA in aquatic environments	48.6
T13	The public does not trust the results obtained by eDNA analysis	45.9
T14	The commercialization of assays by private laboratories poses an ethical problem for the adoption of eDNA	44.7
T15	eDNA analysis protocols change too often to allow for long-term monitoring	42.1
T16	Approaches based on eDNA are perfectible, which compromises their adoption	34.2
T17	Universities and governments are not responsible for the dissemination of available primers	29.7
T18	Climate change compromises the accuracy and relevance of reference databases	18.9
T19	Climate change could make eDNA analysis less reliable	13.5
T20	Taking eDNA samples can damage natural environments	10.5

An open-ended question allowed respondents to identify what could hinder the adoption of eDNA by new end-users. Overall, the respondents indicated that the lack of knowledge regarding the limitations and potential of eDNA may lead to inappropriate protocol selection and implementation, resulting in misinterpretation of results and, ultimately, failure that could foster skepticism toward the method. More specifically, nine threats to adoption by new end-users were identified by the respondents: lack of knowledge about methods and lack of comprehension of the results (n = 14), limited organizational expertise and lack of training (n = 8), lack of standards and norms (n = 7), complexity of protocol implementation (n = 5), misuses and confounding data (n = 4), cost of equipment and analysis (n = 3), unfavorable regulations (n = 2), and limited access to laboratories (n = 2).

The respondents were also asked about potential levers to facilitate the adoption of eDNA. Four main levers were identified: offering training to demonstrate the usefulness and effectiveness of eDNA, but also to increase the capacity of end-users (n = 6), strengthening the capacity and scope of eDNA (n = 5), increasing the number of laboratories (n = 5), and extending access to complete mitogenome sequences across species (n = 2).

### What about trust in eDNA analysis results?

Regardless of where they came from, or how many years of experience they had, 52.6% of the respondents expressed mistrust toward eDNA-based analyses conducted by citizens, and 26.3% reported a lack of trust in the results produced by municipal authorities (Table 5). In their comments, they mentioned that their lack of trust was because some citizens or municipal authorities might not have the right training to properly use eDNA, or that they might try to bias the results to push forward a political agenda.

**Table 5:** A eDNA expert agreement rates with statements related to trust in eDNA analyses generated from interviews with end-users

Survey statement	Agreement with statement (% of total respondents)
I do not trust the results of eDNA-based analyses conducted by citizens	52.6
I do not trust the results of eDNA-based analyses conducted by municipalities	26.3
I do not trust the results of eDNA-based analyses conducted by private companies	18.4
I do not trust the results of eDNA-based analyses conducted by non-governmental organizations (NGOs)	18.4
I do not trust the results of eDNA-based analyses conducted by government ministries	7.9
I do not trust the results of eDNA-based analyses conducted by universities	7.9

## Discussion

Our SWOT analysis highlighted key contrasts in how eDNA experts and end-users perceive eDNA-based monitoring methods. Both groups acknowledged key strengths—such as eDNA’s high sensitivity and precision, its non-intrusive nature, its utility as a decision-support tool, and its suitability for sampling in remote or hard-to-access areas—which are consistent with those previously reported in the literature (Beng & Corlett, 2020; Evans & Lamberti, 2018; Ruppert et al., 2019). Despite these advantages, eDNA experts agreed with end-users about clear weaknesses such as inability to assess organism health, biomass, or abundance, lack of comprehensive reference libraries (Keck et al., 2022), and potential for misuse by untrained end-users. As for the strengths, the weaknesses also corresponded with those reported in the literature (Farrell et al., 2021; Iacaruso et al., 2025; Keck et al., 2022).

Agreement between experts and end-users was much higher on strengths and opportunities (all but one statement with > 60% agreement) than on weaknesses and threats (respectively 55% and 40% of statements with > 60% agreement). For both weaknesses and threats, about a quarter of the statements had less than 40% agreement. Interestingly, more statements received 40–60% of agreement for threats (35% of statements) than for weaknesses (17%). These statements with intermediate agreement are deemed to represent disagreement between experts, with some leaning on the side of end-users, and others not.

These results may indicate that the relative advantages of eDNA are widely acknowledged, which suggests that they have been effectively communicated to and assimilated by end-users. This could also suggest a bias towards genomics (Hochschild & Sen, 2015), or more generally a “technology effect” (sensu Clark et al. (2016)) given that technology or innovation is often associated with progress. Considering the risks linked to the misuse and misinterpretation of eDNA results, as well as the lack of consensus among experts regarding its weaknesses, it is essential to critically examine how the message is conveyed. Emphasizing strengths without adequately addressing limitations may lead to unmet expectations, foster user dissatisfaction, undermine trust, and ultimately contribute to the rejection of the innovation.

Some end-users expressed concerns about the potential misuse of eDNA. Importantly, these concerns did not stem from a lack of understanding but from a clear awareness of technology’s capabilities and risks. As highlighted by Fernandez and Stewart (2025), informed end-users – precisely because they understood its power – fear that without proper safeguards, eDNA could be misapplied or fail to fill their needs. Such cases highlight that the issue is not simply one of “educating” end-users; rather, it requires meaningful dialogue and the establishment of ethical frameworks to ensure that these risks do not materialize.

### Disagreements or contradictions regarding the use of eDNA

Regarding the differences between eDNA experts and end-users in their perception of eDNA weaknesses, we observed that experts expressed confidence in end-users’ organizational capacity to implement DNA testing. However, this perception contrasts with operational limitations reported by end-users. For instance, only 39.5% of experts agreed that applying an experimental design is too complex for non-scientists, and only 21.6% agreed that sample handling, transport, and storage are too complex for non-scientists. These relatively low agreement levels indicate that experts consider these steps manageable, whereas end-users often perceive them as significant barriers. Similarly, eDNA’s potential to engage non-scientists and promote broader participation was widely recognized as an opportunity yet also raised concerns under threats and weaknesses, particularly regarding data quality, user training, and misinterpretation of its capabilities. While eDNA is portrayed as easy to collect, respondents agreed that its effective implementation ultimately relies on specialized laboratory expertise and adequate training. Experts acknowledged the intricacy of protocols yet expressed confidence in non-specialists’ ability to carry out sample collection. Our findings align with those of Mathieu et al. (2020) who noted that while field sampling can be reliably conducted by non-specialists, the downstream analytical processes remain technically demanding, particularly for metabarcoding approaches. This duality underscores the importance of robust training and standardized protocols to mitigate risks associated with broader participation, such as data misinterpretation and quality concerns (Dickie et al., 2018; Mathieu et al., 2020; Zinger et al., 2019).^1^

While many respondents acknowledged the potential of eDNA as a standalone method, others expressed more caution. This divergence likely stems from variations in individual experience, regulatory environments, and levels of trust in emerging technologies, as well as the length of time since the technology was adopted within each jurisdiction (Gupta et al., 2012; Venkatesh & Davis, 2000). Importantly, whether eDNA should serve as a primary tool or remains complementary ultimately depends on the specific research or management question being addressed (Iacaruso et al., 2025; Kelly et al., 2017). Ambivalence in some responses suggests a prudent reluctance to over rely on eDNA or to prematurely replace conventional approaches. The weaknesses temper the strengths and demonstrate just how far we have to go to fulfill the promise of an approach that would save both time and money, while meeting end-users’ needs (e.g., biomass, abundance, health conditions).

### The role of trust in adopting innovation

Widespread adoption of eDNA-based biodiversity monitoring methods is hindered not merely by lack of knowledge, skills, or access, but rather—as evidenced by our comparison between experts and end-users—by issues tied to trust. This trust deficit pertains not only to the approach itself, but also to inter-organizational relationships and structural limitations (Bachmann et al., 2015). Although enhancing knowledge may improve perceived usefulness, and experimentation may foster perceptions of ease of use (Rogers, 2003; Thivierge et al., 2025), knowledge transfer alone is insufficient to resolve trust-related barriers between organizations (De Wit-de Vries et al., 2019).

Trust in eDNA-based methods encompasses multiple dimensions: public trust in the method’s validity; expert trust in results produced by non-specialists or citizen scientists; trust among researchers in the consistency and reliability of results produced by other laboratories, and trust among different groups of end-users. Lack of trust may stem from one or both of two reasons: some believe certain groups will be unable to conduct sampling or analyses properly, while others worry that results could be used to serve a hidden agenda. As Beng & Corlett (2020) and Fernandez & Steward (2025) noted, concerns about misuse often stem from informed awareness of eDNA’s power – not ignorance. This is especially evident among NGOs and Indigenous communities, whose skepticism toward government or industry-led eDNA initiatives reflects power dynamics and historical experiences (Arbour & Cook, 2006; Gray et al., 2017; van Holst Pellekaan, 2000).

It is worth revisiting the issue of trust between private laboratories and academic institutions, as each questions the other's rigor throughout the analytical and interpretative process. Inter-laboratory differences in eDNA extraction performance and detection success have been documented, underscoring the need for standardized and transparent protocols (Rodriguez et al., 2025). A shared concern regarding the validity of results emerges among both experts and end-users. However, this concern takes a different form among end-users, who fear that results may be manipulated or misused, particularly given the indirect nature of the observational approach. While experts place their trust in the method when protocols are rigorously applied within a sound experimental design, field actors tend to rely more on their own judgment and conventional approaches to obtain the information they seek. In this context, standardized protocols may foster trust among experts from different backgrounds, but not necessarily among end-users, who must rely on laboratories’ interpretations without having the expertise to assess their validity.

While the surveyed experts underscored the importance of standardized protocols, those presently under development appear to fall short of expectations. This may be in part due to the need for flexibility in methodology while having some fundamental consensus on performance characteristics. The CSA standards represent a notable attempt to provide performance and minimum reporting requirements as a starting point. However, the tepid endorsement of the CSA standards’ capacity to ensure result validity revealed in our results is indicative of the difficulty of attaining standardization. Although Institutional and regulatory recognition can help legitimize eDNA methods (Bernos et al., 2023), our findings suggest that trust (in eDNA) will also be built through end-users’ direct experimentation in the field, validating results against their own observations (Thivierge et al., 2025).

## Conclusion

This study presents the first SWOT analysis of eDNA in Canada that integrates end-users’ perceptions and experts’ insights. By comparing expert and end-user perspectives, our SWOT analysis revealed technical and operational gaps as well as relational challenges that influence the adoption of eDNA-based biodiversity monitoring methods. Trust emerged as a critical factor because it shapes how actors perceive the validity of results, the rigor of protocols, and the intentions behind their use. It is important to note that the gap identified in this study reflects a lack of trust rather than a lack of confidence: end‑users generally understand the strengths and limitations of eDNA, and addressing their concerns requires more than training or increased technical exposure. Minimizing the issue of weak eDNA uptake by treating it solely as a confidence gap risks overlooking the relational and institutional dimensions highlighted by our results. Several of the identified weaknesses reflect unmet needs among end-users, for whom the added value of eDNA-based biodiversity monitoring methods may not seem straightforward as long as conventional approaches remain necessary to fully address their needs. Expanding the adoption of eDNA among environmental professionals will therefore require that the scientific community aligns more closely with end-users’ realities. Building trust cannot rely solely on methodological standardization. It also depends on transparent governance, knowledge sharing, and the implementation of mechanisms that ensure accountability across institutional boundaries. Addressing these dimensions is essential for moving eDNA from a promising innovation to a widely accepted environmental monitoring tool.

## Data Availability

The interview data supporting this study cannot be shared publicly as they contain information that could compromise the privacy of respondents. In accordance with ethical requirements, these data are available from the corresponding author upon reasonable request and subject to confidentiality agreements. However, the anonymized survey data generated and analyzed during the study are available upon request from the corresponding author.

## Footnotes

1 Unlike qPCR-based eDNA assays, which benefit from validated protocols (e.g., CSA standards) and streamlined downstream analysis suitable for institutional uptake (Langlois et al., 2021, 2025), (metabarcoding lacks standardized workflows. No validated protocols currently exist — nor are expected in the near future — and the sequencing output is much more complex for downstream processing than for qPCR-based eDNA assays.
